# Stabilized Palladium Nanoparticles from Bis-(*N*-benzoylthiourea) Derived-Pd^II^ Complexes as Efficient Catalysts for Sustainable Cross-Coupling Reactions in Water

**DOI:** 10.3390/molecules29051138

**Published:** 2024-03-04

**Authors:** Samet Poyraz, H. Ali Döndaş, Samet Belveren, Senanur Taş, Raquel Hidalgo-León, José Trujillo-Sierra, Lesly V. Rodríguez-Flórez, Mª de Gracia Retamosa, Ana Sirvent, Mohammad Gholinejad, Sara Sobhani, José M. Sansano

**Affiliations:** 1 Department of Basic Pharmaceutical Sciences, Faculty of Pharmacy, Çukurova University, Balcalı, Adana 01330, Türkiye; s.poyraz88@gmail.com (S.P.); bvrnsamet@mersin.edu.tr (S.B.); 2 Department of Biotechnology, Institute of Natural and Applied Sciences, Çukurova University, Balcalı, Adana 01330, Türkiye; senanurtas99@gmail.com; 3 Departamento de Química Orgánica, Instituto de Síntesis Orgánica (ISO), Centro de Innovación en Química Avanzada (ORFEO-CINQA), University of Alicante, P.O. Box 99, 03690 Alicante, Spain; raquel.hidalgo@ua.es (R.H.-L.); josetrujillosierra98@gmail.com (J.T.-S.); lesly.rodriguez11@ua.es (L.V.R.-F.); graciretamosa@hotmail.com (M.d.G.R.);; 4 Department of Chemistry, Institute for Advanced Studies in Basic Sciences (IASBS), P.O. Box 45195-1159, Zanjan 45137-66731, Iran; gholinejad@iasbs.ac.ir; 5 Research Center for Basic Sciences & Modern Technologies (RBST), Institute for Advanced Studies in Basic Sciences (IASBS), Zanjan 45137-66731, Iran; 6 Department of Chemistry, College of Sciences, Shiraz University, Shiraz 71454, Iran; ssobhani@birjand.ac.ir; 7 Department of Chemistry, College of Sciences, University of Birjand, Birjand 9717434765, Iran

**Keywords:** nanoparticles, palladium, Suzuki, Heck, Hiyama, Buchwald–Hartwig, Hirao, Sonogashira, water, heterogeneous catalysis

## Abstract

Stable palladium (II) complexes, incorporating a double (*N*-benzoylthiourea) arrangement bonded to a complex heterocyclic scaffold, are used as precursors of catalytic species able to promote Suzuki–Miyaura, Mizoroki–Heck, Hiyama, Buchwald–Hartwig, Hirao and Sonogashira–Hagihara cross-coupling transformations in water. These sustainable processes are chemoselective and very versatile. The nanoparticles responsible for these catalytic reactions were analyzed and studied. Their usefulness is demonstrated after several tests and analyses. The heterogeneous character of this species in water was also confirmed.

## 1. Introduction

The element Palladium constitutes a crucial component in many scientific areas and industrial processes [1]. Due to its low presence in the ore deposits around the world, the synthesis of palladium nanoparticles (PNPs) represents an extraordinary advance. It is very well known that there is a larger surface area exhibited by PNPs rather than palladium bulk metal. Concerning this, automobile sector, electronics, catalysis, dentistry, jewelry and biomedical therapies for cancer take advantage of this property to expand the applications [1]. In the field of the catalysis, the carbon–carbon bond-forming reactions represent a very exploited and useful tool in general organic synthesis [2,3]. Implementing the concept of PNPs in these transformations, the term green process can be associated with them [4]. Thus, for example, Kumada–Tamao–Corriu [5], Mizoroki–Heck [6], Negishi [7], Stille [8], Sonogasira–Hagihara [9], Suzuki–Miyaura [10,11], Hiyama [12], Hirao [13] couplings, Buchwald–Hartwig reaction [13], etc., have been published using PNPs in a green manner. Another important detail in this field is the procedure employed to prepare PNPs (physical or chemical). From the industrial point of view, the physical methods require large energy costs due to the high temperatures and/or pressures involved. The chemical methods usually require harmful solvents and hazardous reducing or stabilizing agents, generating toxic by-products [1]. Thus, electrochemical, sonochemical, sol-gel, supercritical fluid, plant/bacteria-mediated methodologies, etc., have been implemented [1].

In this work, we will survey the ability to generate stabilized PNPs in the reaction media from already-known palladium(II) complexes, testing their efficiency in sustainable cross-coupling reactions such as Suzuki–Miyaura, Mizoroki–Heck, Sonogashira–Hagihara, Hirao, Hiyama couplings and Buchwald–Hartwig reactions in water [14]. The characterization of the PNPs prepared in situ, as well as their recyclability, will also be analyzed.

## 2. Results and Discussion

### 2.1. Suzuki–Miyaura Cross-Coupling

**Figure 1:**
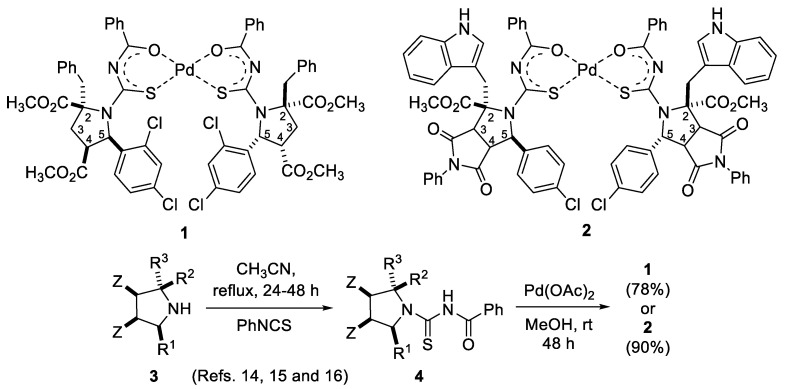
Structure of the palladium(II) complexes (**1** and **2**) precursors of PNPs and their preparation.

Using the known methodology developed by our group, the amphiphilic palladium complexes **1** and **2** were obtained (Figure 1). For this purpose, the starting *endo*-prolinates **3**, isolated after 1,3-dipolar cycloaddition [15,16,17], were allowed to react with phenyl isothiocyanate in refluxing acetonitrile to obtain compounds **4** in good yields. Finally, chelation was achieved using palladium(II) acetate in methanol at rt for 48 h. *Cis*-complexes **1** and **2** were isolated in yields depicted in Figure 1. The relative configuration of complex **1** was unambiguously determined according to an X-ray diffraction pattern observed in a preceding work [18]. It is characterized and evaluated as a potential anti(myco)bacterial and antifungal agent [18,19]. The structure of complex **2** was deduced according to NMR experiments. These two aggregates are very stable and can be stored for a long time under argon atmosphere [20,21]. They were evaluated to a classical Suzuki–Miyaura cross-coupling, keeping in mind the aim to complete a sustainable process. In this reaction, and that described in the manuscript, the stoichiometry of the reagents was briefly optimized but was always based in our previous contributions.

**Scheme 1:**
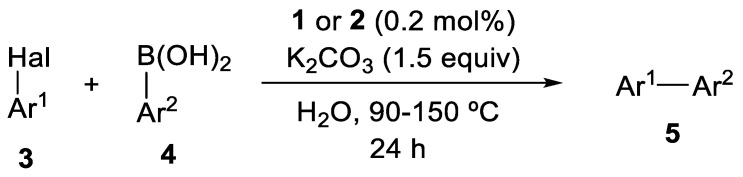
Suzuki–Miyaura cross coupling promoted by complexes **1** or **2**.

The first idea was using water as a solvent under standard conditions, set up by our group in a previous contribution [22]. The optimized reaction between 4-iodoanisole and phenylboronic acid operated at 90 °C using potassium carbonate as a base and 0.2 mol% of the catalyst for 24 h (Scheme 1). The best precursor of the palladium catalytic species was complex **1** rather than complex **2** (compare entries 1op and 2op of Table 1). The influence of the solvent was analyzed; thus, when the reaction was performed in toluene, 1,4-dioxane and DMF, the chemical yields did not improve compared with the yield obtained using water (entries 1op and 3op-5op of Table 1). The addition of 0.1 mol% palladium complex did not offer high yields (Table 1, entry 6op), and the lowering of the temperature was unfruitful (Table 1, entry 7op). The reaction did not occur at all in the absence of the pre-catalyst. Electron-donating and electron-withdrawing groups, bonded to iodoarene **3**, allowed for the reaction in very good yields (Table 1, entries 1op and 8–11). The carbon–chlorine bond remained intact after the reaction shown in entry 11 of Table 1. The Suzuki–Miyaura coupling (Scheme 1) between iodobenzene and different arylboronic acids afforded biaryls **5** in high yields (Table 1, entries 12 and 13). The reaction of aryl bromides needed 120 °C to obtain the same range of yields than the corresponding iodoarenes (Table 1, entries 14–16). However, chloroarenes **3** reacted slowly and required higher temperatures (150 °C) to obtain moderate to good chemical yields of **5** (Table 1, entries 17–19).

### 2.2. Mizoroki–Heck Reaction

**Scheme 2:**
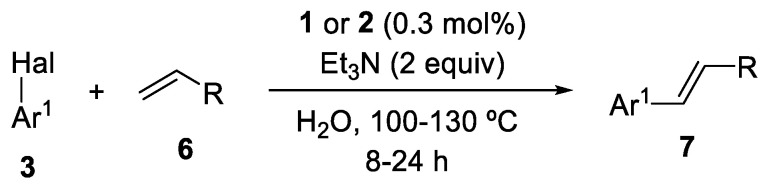
Mizoroki–Heck reaction promoted by complexes **1** or **2**.

Having the results of the previous coupling reaction, the Mizoroki–Heck reaction in water (Scheme 2) was next surveyed employing a set of conditions implemented in our group [23]. In this manner, the optimized reaction between 4-iodoanisole (**3a**) and *n*-butyl acrylate (**6a**) operated at 100 °C using triethylamine as a base, and 0.3 mol% of the catalyst for 8 h was performed. The best precursor of the palladium catalytic species was again complex **1** (Table 2, entries 1op and 2 op). Toluene, 1,4-dioxane and DMF afforded similar chemical yields to the reaction run with water, but the ^1^H NMR spectra of these three crude products were not very clean (Table 2, entries 3op, 4op and 5op). Potassium carbonate afforded similar results (Table 1, entry 6op), whilst sodium hydroxide was not a suitable base for this process (Table 1, entry 7op). Lowering of the catalyst loading (0.2 mol%) and the temperature of the reaction (90 °C) furnished lower yields of the expected alkene **7** (Table 1, entries 8op and 9op). The combination of assorted iodoarenes **3** and *n*-butyl acrylate (**6a**) or styrene (**6b**) produced compounds **7** in high isolated yields (Table 1, entries 1op and 10–14). Again, the chlorine atom remained intact in the reaction shown in entries 13 and 14 of Table 2. Bromobenzene and *p*-bromoanisole reacted with *n*-butyl acrylate (**6a**) or styrene (**6b**) at 120 °C in 8 h (Table 2, entries 15–18). This higher temperature was required to obtain almost complete conversions. However, the reaction of chlorobenzene was very difficult to achieve. In this case, moderate chemical yields of **7c** and **7d** were isolated (50 and 54%, respectively) using 130 °C and 24 h of reaction (Table 2, entries 19 and 20).

### 2.3. Hiyama Cross-Coupling

**Scheme 3:**
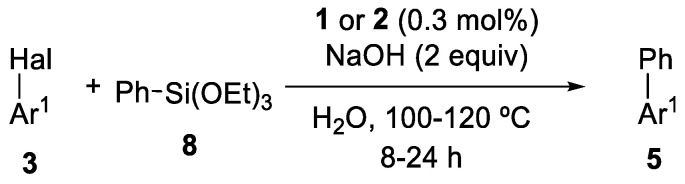
Hiyama cross-coupling promoted by complexes **1** or **2**.

To probe the catalytic activity of the palladium active species toward the Hiyama cross-coupling reactions (Scheme 3), the reaction of iodoanisole (**3a**) and triethoxyphenylsilane (**8**) in aqueous media was chosen as a model reaction [23]. Various reaction parameters were rapidly screened, obtaining the following results summarized in Table 3: (a) the efficiency of the catalyst precursor **1** was higher than the analogous one obtained for the catalytic species precursor **2** (Table 3, compare entries 1op and 2op); (b) toluene, 1,4-dioxane and DMF afforded similar chemical yields to the reaction run with water, but the ^1^H NMR spectra of these three crude products were not very clean (Table 3, entries 3op, 4op and 5op); (c) the most appropriate base was NaOH rather than potassium carbonate or triethylamine (Table 3, entries 6op and 7op); (d) the optimal operation temperature was 100 °C (Table 3, entry 8op); (e) and the suitable catalyst loading was generated in the presence of 0.3 mol% of **1** (Table 3, entry 9op). In general, iodoarenes reacted at 100 °C (Table 3, entries 1op and 10–12) and bromoarenes at 110 °C (Table 3, entries 13–15), achieving chemical yields in the same range (80–75%). Despite using 130 °C and reaction times of 24 h, the yields of product **5** were moderate when aryl chlorides were used as starters (Table 3, entries 16–18).

### 2.4. Buchwald-Hartwig Cross-Coupling

**Scheme 4:**
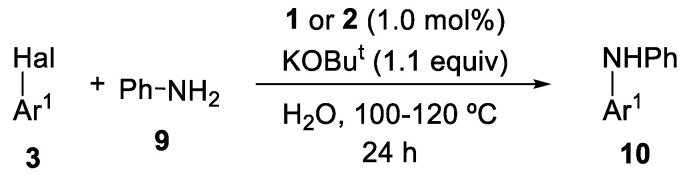
Buchwald–Hartwig cross-coupling promoted by complexes **1** or **2**.

The catalytic activity of the species studied before was assessed in the Buchwald- Hartwig reaction (Scheme 4) following the established conditions in water detailed in the literature [24,25]. The conditions displayed in entry 1op of Table 4 were achieved using the catalyst precursor **1**, 4-iodoanisole and aniline [26]. The 41% yield could be improved neither by the presence of the pre-catalyst **2** nor by increasing the temperature (compare entries 1op–3op of the Table 4). The employment of potassium phosphate as a base, lower temperatures than 100 °C, and a catalyst loading of 2 mol% were not appropriate (Table 4, entries 4op–6op). Toluene, 1,4-dioxane and DMF did not improve the yield obtained in the reaction run with water. The reaction offered modest yields with iodoarenes (41–58%, Table 4, entries 1op, 7 and 8), but the scope was not so wide as it was described in the previous coupling transformations. In fact, 4-bromoanisole afforded a very low yield after reaction with aniline at 120 °C for 24 h (Table 4, entry 9).

### 2.5. Hirao Cross-Coupling

**Scheme 5:**
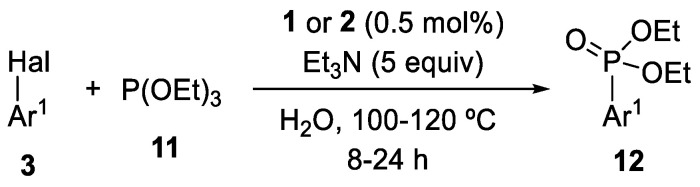
Hirao cross-coupling promoted by complexes **1** or **2**.

A Hirao cross-coupling reaction (Scheme 5) means the formation of a C(sp^2^)-P bonds catalyzed by transition metals [27], and it allows us to obtain arylphosphonates **12**, which are valuable intermediates and molecules in organic synthesis [23]. The reaction between triethylphosphite (**11**) and 4-iodoanisole (**3a**) was chosen as a model reaction to find the best suitable reaction conditions. Initially, the reaction was performed with the catalyst and triethylamine in water. The efficiency of the precatalyst **1** was higher once more, yielding **12a** in 88% (compare entries 1op and 2op of the Table 5). Other solvents tested were not as effective as water (Table 5, entries op3-op5). Triethylamine was the most appropriate base (compare entries 1op and 6op and 7op of the Table 5), and the reaction did not operate completely at 90 °C after 8 h (Table 5, entry 8op). A lower amount of the catalyst loading was not beneficial for the full transformation (Table 5, entry 9op). Iodoarenes reacted satisfactorily under the optimal conditions defined in entry 1op of the Table 5 (entries 1op, 10 and 11 of the same Table) as did bromoarenes, but afforded slightly lower chemical yields (Table 5, entries 12–14). Aryl chlorides also gave very interesting results of products **12,** although using higher temperatures (120 °C) for 24 h (Table 5, entries 15–17).

### 2.6. Sonogashira–Hagihara Cross-Coupling

**Scheme 6:**
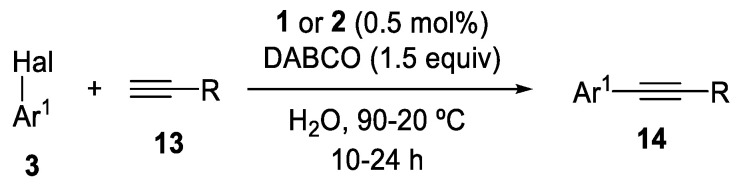
Sonogashira–Hagihara cross-coupling promoted by complexes **1** or **2**.

The Sonogashira–Hagihara cross-coupling reaction (Scheme 6) was next assessed using these two palladium catalyst precursors. Using the parameters displayed by our group [28], the optimization was performed with phenylacetylene (**13a**) and 4-iodoanisole (**3a**). The employment of 90 °C, DABCO as a base in water for 10 h afforded compound **14a** in 91% yield in the presence of the catalyst generated by **1** (Table 6, entry 1op). However, an 81% yield was achieved when complex **2** was used as a pre-catalyst (Table 6, entry 2op). Using toluene, 1,4-dioxane, or DMF, similar yields were obtained (Table 6, entries op3–op5), but water was selected for operational simplicity and further recycling of the catalytic suspension. DABCO as a base gave the best results rather than other bases such as triethylamine or potassium carbonate (compare entries 1op, 6op and 7op of the Table 6). Lower temperature and catalyst loadings were also not productive for the reaction completion at the same reaction time (Table 6, entries 8op and 9op). Iodoarenes reacted with phenylacetylene (**13a**) or propargyl alcohol (**13b**), furnishing the corresponding alkynes **14** in very good yields (Table 6, entries op1 and 10–14). Also, good yields were obtained employing bromoarenes at the same conditions with these two different terminal alkynes **14a** and **14b** (Table 6, entries 15–18). Aryl chlorides were demonstrated to be good starting materials, obtaining results similar to the obtained ones for the reactions performed with bromides but while heating the reaction to 120 °C for 24 h (Table 6, entries 19–22).

### 2.7. Characterization of the Catalyst and Study of Their Separation and Recycling

**Figure 2:**
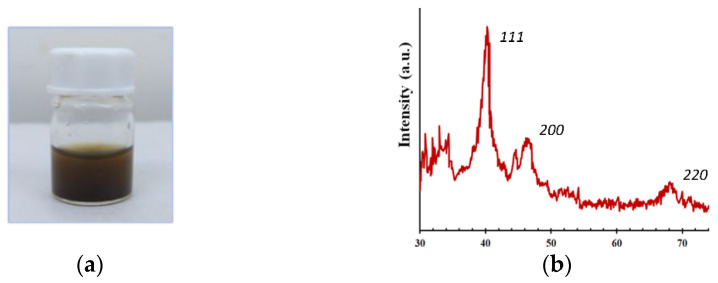
(**a**) Nanoparticle suspension in water studied in this section. (**b**) XRD pattern of the intensity of the signal of nanoparticles versus 2θ angle (°).

**Figure 3:**
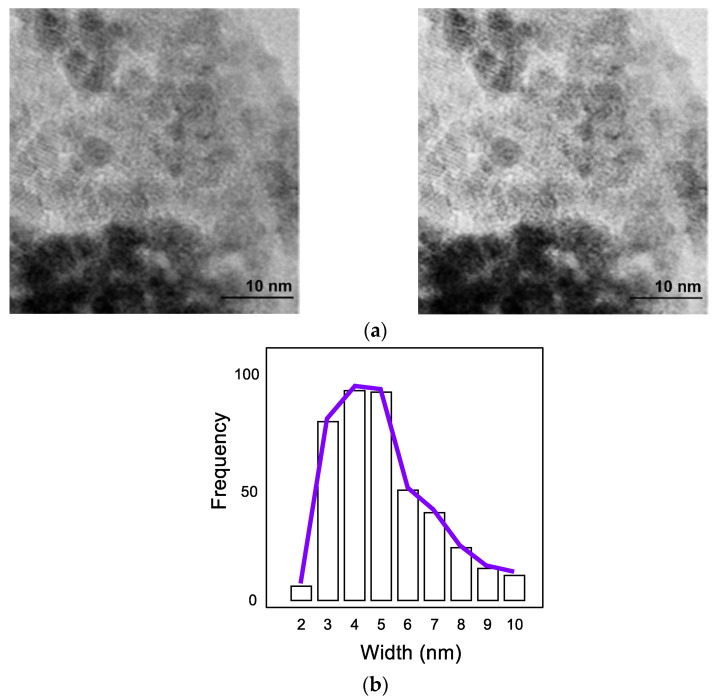
(**a**) TEM image of the nanoparticles isolated after the first cycle of the Suzuki–Miyaura/Hirao cross coupling transformation. (**b**) Estimated size distribution of the nanoparticles according to TEM histogram.

Complexes **1** or **2** were presumably reduced during all the experiments with palladium(0) by the cooperative effect of all reagents and additives present in the aqueous reaction media under light exposure. Thus, for example, Suzuki–Miyaura and Mizoroki–Heck cross coupling reactions were successfully promoted by freshly generated nanoparticles under these particular conditions in the absence of phosphines [29]. A brief analysis of the morphology of the catalytic species was performed. Focusing on the Suzuki–Miyaura and Hirao reactions catalyzed by adding complex **1**, once the transformations were finished, the aqueous suspensions (Figure 2a) were washed twice with ethyl acetate, and the water was slowly evaporated under reduced pressure and then completely dried under vacuum at 60 °C. The XRD patterns of finely dispersed powder showed that the main bands of the generation of Pd^0^ species at (111), (200), (220) and (311) crystallographic planes were detected at 40.2°, 46.7° and 68.2° (Figure 2b) [30]. The XRD patterns for the Pd nanoparticles showed a wide peak centered at around 40.35 °. The TEM image (Figure 3) and the internal measurements revealed a particle size ranging between 5.0 and 3.5 nm, which is in accordance with the estimated value using the Scherer model (The average size of the Pd nanoparticles (4.8 nm) was calculated from the overall width at half maximum of the strongest diffraction peak {111} using the Scherer equation. Please, see Ref. [22]) [28].

**Figure 4:**
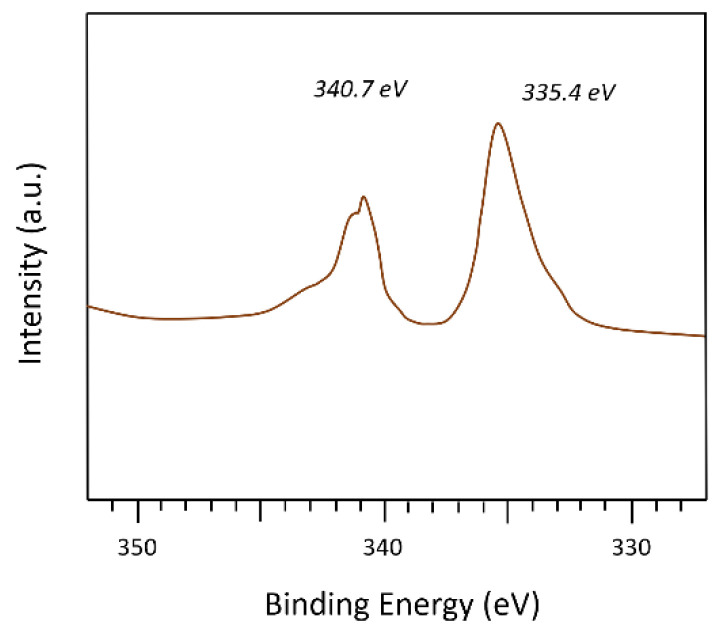
XPS analysis of the nanoparticles obtained after the first Suzuki–Miyaura/Hirao cross coupling transformations.

The presence of the palladium(0) in these nanoparticles was also confirmed by XPS analysis (Figure 4), showing intensities corresponding to Pd^0^ 3d_3/2_ and 3d_5/2_ peaks centered at 335.4 and 340.7 eV [30].

The composition of the catalytic species was determined using XPS, ICP and elemental analysis (EA) of three different samples for each transformation (both Suzuki-Miyaura and Hirao reactions, Table 7). According to all of the limitations of each instrumental techniques and the complementary character of them, it is possible to conclude that values of the surface shown by XPS are very closed to the EA and ICP ones. The range of palladium composition is 98.9–98.5%, which is contaminated with small amounts of carbon, hydrogen, nitrogen and sulfur, but no traces of chlorine were detected (see Table 7).

With the aim to extract an accurate and reliable result given by the mercury test, we followed the recommendations and suggestions published in the literature [31]. Thus, keeping in mind the colloidal aspect of the nanoparticles in water obtained at the end of the reaction (Figure 2a), the addition of mercury (500 equivalent relative to the palladium loading) onto a freshly set Suzuki–Miyaura cross coupling a 300 rpm, under the optimized conditions detailed in entry 1op of the Table 1, after 24 h at 90 °C, a 39% yield of **5a** was isolated.

Another experiment performed with the colloidal nanoparticles in water described before (Figure 2a), was the filtration of this suspension at 90 °C (hatman Paper for Quantitative Analysis 1450-110 was employed with a nominal particle retention of 2.7 µm) and immediately tested in the Suzuki–Miyaura coupling. In this experiment (entry 1op of the Table 1), the result of the isolated **5a** was 44% yield. So, a heterogeneous system in water can be confirmed employing the metal complex **1** under these specific conditions.

**Figure 5:**
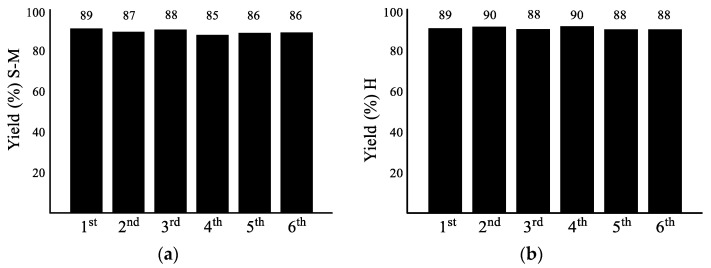
Recycling study of the catalyst in (**a**) the Suzuki–Miyaura cross-coupling and (**b**) Hirao cross coupling.

Continuing with this suspension of nanoparticles in Figure 2a, the recycling of them in the two selected transformations (Suzuki–Miyaura and Hirao) was assessed (Figure 5). The chemical yields of both transformations maintained the general range along the six cyclic batches analyzed in the two plots of the Figure 5. Two recycling experiments were consecutively run in the Mizoroki–Heck, Hiyama, Buchwald–Hartwig, and Sonogashira–Hagihara cross-couplings, obtaining 83 and 84%, 81 and 80%, 41 and 41%, 91 and 92% yields, respectively.

**Figure 6:**
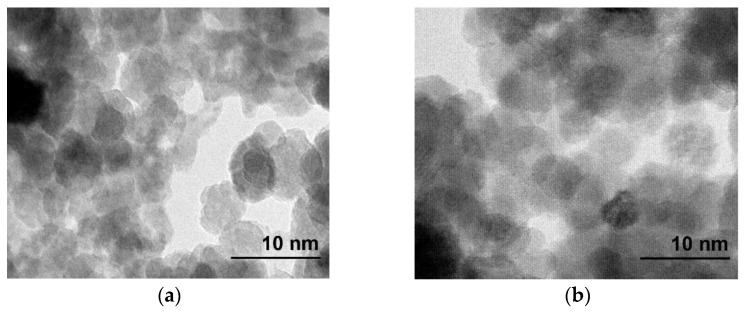
TEM image of the nanoparticles isolated after the fifth cycle in (**a**) Suzuki–Miyaura and (**b**) Hirao cross coupling transformations.

TEM images of the catalyst system were also analyzed after the sixth reaction cycle/batch (Figure 6). On them, an increment of the particle size was detected but maintained very small diameters in some nanoparticles, as can be seen in parts of the images; that means the distribution range was wider as a consequence of a small sinterization process along the series of repetitive cycles (Figure 6a,b).

## 3. Materials and Methods

### 3.1. General

All reagents and solvents were commercially employed and used without further purification. The aldehydes were distilled under reduced pressure prior to use. Analytical TLC was conducted on Schleicher and Schuell F1400/LS 254 silica gel plates, and the different compounds distributed along the plate were visualized with UV light (λ = 254 nm). Flash chromatography was performed on hand-packed columns of Merck silica gel 60 (0.040–0.063 mm). ICP-MS analysis was carried in an Agilent-7700x (ICP-MS) apparatus. XRD studies were conducted in a Bruker D8-Advance with an X-ray tube cathode Cu K α, and a 3D Pixcel detector. NMR spectra were obtained using a Bruker AC-300 or AC-400 and were recorded at 300 or 400 MHz for ^1^H NMR and 75 or 100 MHz for ^13^C NMR, using CDCl_3_ as the solvent and TMS as internal standard (0.00 ppm) unless otherwise stated. The following abbreviations are used to describe the signal patterns: s = singlet, d = doublet, t = triplet q = quartet, m = multiplet or unresolved and br s = broad signal. Coupling constants (*J*) are given in Hz, and chemical shifts in ppm. ^13^C NMR spectra were referenced to CDCl_3_ at 77.0 ppm. The ultrasound bath employed was Argo Lab AU-32, and the centrifuge was Hettich Zentrifugen (universal 320). The TEM images were recorded on a microscope JEOL model JEM-2010. XPS analyses were performed using a VG-Microtech Multilab 3000 spectrometer, equipped with an Al anode and on a K-Alpha Thermo-Scientific spectrometer.

### 3.2. General Procedure for the Preparation of the Pd(II) Complexes

The palladium complexes **1** and **2** were prepared by modifying the procedure found in the literature [18,19]. Thus, palladium acetate (113 mg, 0.5 mmol) dissolved in methanol (15 mL) was added dropwise to the *N*-benzoyl thiourea **4** as a ligand (1 mmol) dissolved in methanol (20 mL) and stirred for 36 h at room temperature. The precipitation was filtered and purified by crystallization from methanol, finally obtaining complexes **1** and **2**.

*Palladium(II) complex* **1** [18]: Brownish yellow solid, 99 mg, 78% yield; mp 187–189 °C (MeOH, decom.). ^1^H NMR (400 MHz) δ: 8.18–8.15 (m, 6H, minor and major), 8.01–7.94 (m, 4H, minor and major), 7.54–7.14 (m, 16H, minor and major), 5.56 (d, *J* = 9.9 Hz, 1H-5, major), 5.48 (d, *J* = 10.1 Hz, 1H-5, minor), 4.28 (d, *J* = 14.0 Hz,1H-6, major), 4.23 (d, *J* = 14.1 Hz, 1H-6, minor), 3.80 (s, 3H, minor), 3.75 (s, 3H, major), 3.36–3.43 (m, 2H, major and minor), 3.22 (s, 3H, minor), 3.21 (s, 3H, major), 2.99–2.91 (m, 2H, major and minor), 2.35–2.28 (m, 2H, major and minor), 2.26–2.16 (m, 2H, major and minor). ^13^C NMR (100 MHz) δ: 173.1 (C=S minor), 173.0 (CS major), 172.2 (CO minor), 172.1 (CO major), 172.0 (CO minor), 172.05 (CO major), 169.7 (CO minor), 169.6 (CO major), 136.5 minor, 136.4 major, 136.3 minor and major, 135.3 major, 135.2 minor, 134.1 major, 134.0 minor, 133.9 minor and major, 132.2 (C major), 130.2 (3C minor), 130.1 minor and major, 130.0 (4C minor), 129.6 minor, 129.5 major, 129.2 (3C major), 129.0 minor, 128.8 major, 128.2 (4C major), 127.9 minor, 127.8 major, 127.6 (2C minor), 73.5 minor, 73.4 major, 64.1 minor, 63.9 major, 53.1 minor, 53.0 major, 51.6 major and minor, 45.9 major, 45.5 minor, 40.1 minor, 40.0 major, 36.8 minor, 36.6 major IR (cm^−1^) ν_max_: 3027, 2948, 1737, 1497, 1396, 1361, 1246, 1101, 701 cm^−1^. MS (ESI) *m/z* (%): 1283 (29), 1282 (46), 1281 (60), 1280 (96), 1279 (M^+^, 62%), 1278 (100), 1277 (81), 1276 (78), 1275 (66). Elemental Analysis required for C_58_H_54_Cl_4_N_4_O_10_PdS_2_: C, 54.5; H, 4.3; N, 4.4; S, 5.0%; found: C, 54.9; H, 4.0; N, 4.6; S, 4.7%.
*Palladium complex* **2**: Brownish-yellow solid, 123 mg, 90% yield; mp 253–255 °C (MeOH, decomp.); IR (cm^−1^) ν_max_: 3417, 3060, 2952, 1790, 1716, 1495, 1391, 1258, 1201, 1168, 1093, 743. δ_H_ (400 MHz, CDCl_3_): 8.39 (s, 2H, N-*H*), 8.20 (d, 4H, *J* = 7.3 Hz, ArH), 7.73 (d, 2H, *J* = 7.8 Hz, ArH), 7.55 (d, 2H, *J* = 7.3 Hz, ArH), 7.51–7.36 (m, 9H, ArH), 7.32–7.26 (m, 9H, ArH), 7.19 (d, 4H, *J* = 7.7 Hz, ArH), 7.10 (d, 2H, *J* = 1.9 Hz, ArH), 6.52–6.45 (m, 4H, ArH), 5.40 (d, 2H, *J* = 11.0 Hz, 5-*H*), 4.52 (d, 2H, *J* = 15.10 Hz, 6-*H*), 3.95 (s, 6H, OC*H_3_*), 3.89 (d, 2H, *J* = 15.00 Hz, 6′-*H*), 3.84 (d, 2H, *J* = 9.5 Hz, 3-*H*), 2.60 (dd, 2H, *J* = 10.8 Hz, 9.1 Hz, 4-*H*). δ_C_ (100 MHz, CDCl_3_): 172.6 (2xC=S), 172.6 (2xC=O), 172.2 (2xC=O), 172.0 (2xC=O), 169.6 (2xC=O), 136.0 (2C), 135.9 (2C), 135.8 (2C), 133.8 (2C), 132.5 (2C), 130.7 (2C), 130.1 (6C), 129.0 (6C), 128.7 (2C), 128.3 (6C), 127.7 (2C), 125.7 (6C), 124.3 (2C), 122.9 (2C), 120.8 (2C), 117.8 (2C), 111.9 (2C), 108.7 (2C), 68.9 (2C), 54.1 (2C), 53.1 (2C), 48.5 (2C), 31.7 (2C), 30.9 (2C). HRMS: calculated for C_74_H_60_Cl_2_N_8_O_10_PdS_2_: 1462.7717, found: 1462.7711. EA calculated for C_74_H_60_Cl_2_N_8_O_10_PdS_2_: C 60.8, H 4.1, N 7.7, S 4.4%; found: C 60.5, H 4.4, N 7.7, S 4.5%.

### 3.3. General Procedure for Suzuki–Miyaura Cross Coupling Reaction

Aryl halide (0.5 mmol), arylboronic acid (0.75 mmol), K_2_CO_3_ (103 mg, 0.75 mmol), complex **1** (1.3 mg, 0.001 mmol, 0.2 mol%) and water (1.5 mL) were introduced in a pressure tube and warmed in an oil bath at the corresponding temperature for 24 h. Progress of the reactions was monitored by GC or TLC. Afterwards, the crude product was extracted using ethyl acetate (3 × 5 mL). The organic layer was dried and evaporated and further purified by column chromatography. The colloidal aqueous suspension was reused in other identical reactions (six different catalytic cycles, see Figure 5a) or employed for the characterization of the nanoparticles. The final compounds **5a**–**f** were isolated and purified by flash chromatography (silica gel) using mixtures of *n*-hexane/EtOAc as eluent. The pure compounds offered matching spectroscopic data with the analogous ones obtained by our group [22].

*4-Methoxy-1,1′-biphenyl* (**5a**) [32]: Isolated 82 mg (89%, Table 1, entry 1op), 80 mg (87%, Table 1, entry 12), 73 mg (79%, Table 1, entry 14), 50 mg (54%, Table 1, entry 17) as colorless solid. M.p. 88–89 °C (*n*-hexane/AcOEt), Lit. 86–87 °C (hexanes) [32]. *R*_f_ = 0.2 (*n*-hexane). ^1^H NMR (400 MHz) δ (ppm): 7.60 (t, *J* = 8.4 Hz, 4H), 7.47 (t, *J* = 7.6 Hz, 2H), 7.36 (t, *J* = 7.3 Hz, 1H), 7.04 (d, *J* = 8.7 Hz, 2H), 3.90 (s, 3H). ^13^C NMR (100 MHz) δ (ppm): 159.2, 140.8, 133.8, 128.8, 128.2, 126.8, 126.7, 114.3, 55.4.
*4-Methyl-1,1′-biphenyl (***5b**) [33]: Isolated 75 mg (89%, Table 1, entry 8), 73 mg (87%, Table 1, entry 13), 68 mg (81%, Table 1, entry 15), 57 mg (68%, Table 1, entry 18) as colorless solid. M.p. 49–50 °C (*n*-hexane/AcOEt), Lit. 50–51 °C (hexanes) [34]. *R*_f_ = 0.6 (*n*-hexane). ^1^H NMR (400 MHz) δ (ppm): 7.65 (dd, *J* = 33.6, 7.6 Hz, 4H), 7.55–7.41 (m, 3H), 7.35 (d, *J* = 7.8 Hz, 2H), 2.50 (s, 3H). ^13^C NMR (100 MHz) δ (ppm): 141.38, 138.5, 137.1, 129.6, 128.8, 127.1, 127.1, 21.2.
*[1,1′-Biphenyl]-3-carbonitrile* (**5c**) [35]: Isolated 79 mg (88%, Table 1, entry 9) as colorless solid. M.p. 39–40 °C (*n*-hexane/AcOEt), Lit. 38–39 °C (hexanes/EtOAc) [36]. *R*_f_ = 0.2 (*n*-hexane). ^1^H NMR (400 MHz) δ (ppm): 7.92 (s, 1H), 7.87 (d, *J* = 7.9 Hz, 1H), 7.69 (d, *J* = 7.7 Hz, 1H), 7.63–7.59 (m, 3H), 7.56–7.46 (m, 3H). ^13^C NMR (101 MHz) δ (ppm): 142.4, 138.9, 131.5, 130.7, 129.7, 129.1, 128.6, 127.1, 118.9, 112.9.
*[1,1′-Biphenyl]-3-carbaldehyde* (**5d**) [37]: Isolated 82 mg (90%, Table 1, entry 10) as colorless solid. M.p. 54–55 °C (*n*-hexane/AcOEt), Lit. 53–54 °C (hexanes/EtOAc) [38]. *R*_f_ = 0.4 (*n*-hexane).V^1^H NMR (400 MHz) δ (ppm): 10.15 (s, 1H), 8.17 (s, 1H), 7.95–7.41 (m, 8H). ^13^C NMR (101 MHz) δ (ppm): 192.4, 142.2, 139.7, 136.9, 133.1, 129.5, 129.0, 128.7, 128.2, 128.0, 127.2.
*4-Chloro-1,1′-biphenyl* (**5e**) [39]: Isolated 85 mg (90%, Table 1, entry 11) as colorless solid. M.p. 78–79 °C (*n*-hexane/AcOEt), Lit. 77–78 °C (*n*-hexane/EtOAc) [40]. *R*_f_ = 0.4 (*n*-hexane). ^1^H NMR (400 MHz) δ (ppm): 7.62 (dd, *J* = 15.3, 7.9 Hz, 4H), 7.49 (ddt, *J* = 20.3, 14.6, 7.3 Hz, 5H). ^13^C NMR (101 MHz) δ (ppm): 140.0, 139.7, 133.4, 128.9, 128.9, 128.4, 127.6, 127.0.
*Biphenyl-4-carbaldehyde* (**5f**) [41]: Isolated 78 mg (80%, Table 1, entry 16) as colorless solid. M.p. 59–60 °C (*n*-hexane/AcOEt), Lit. 59–60 °C (*n*-hexane/EtOAc) [36]. *R*_f_ = 0.3 (*n*-hexane). ^1^H NMR (400 MHz) δ (ppm): ^1^H NMR (400 MHz, Chloroform-*d*) δ (ppm): 10.15 (s, 1H), 8.17 (s, 1H), 7.92 (m, 2H), 7.68 (m, 3H), 7.50 (dt, *J* = 31.1, 7.3 Hz, 3H). ^13^C NMR (101 MHz) δ (ppm): 192.0, 147.2, 139.7, 135.2, 130.3, 129.0, 128.5, 127.7, 127.4.
*[1,1′-Biphenyl]-4-carbonitrile* (**5g**) [42]: Isolated 63 mg (70%, Table 1, entry 19) as colorless solid. M.p. 85–86 °C (*n*-hexane/AcOEt), Lit. 85–86 °C (*n*-hexane/EtOAc) [43]. *R*_f_ = 0.4 (*n*-hexane). ^1^H NMR (400 MHz) δ (ppm): 7.77 (q, *J* = 8.2 Hz, 4H), 7.69–7.44 (m, 5H). ^13^C NMR (101 MHz) δ (ppm): 145.7, 139.2, 132.6, 129.1, 128.7, 127.7, 127.2, 119.0, 110.9.

### 3.4. General Experimental Procedure for Mizoroki-Heck Reaction

Aryl halide (0.5 mmol), olefin (0.55 mmol), triethylamine (0.14 mL, 1 mmol), H_2_O (1.5 mL) and **1** (1.9 mg, 0.0015 mmol, 0.3 mol%) were introduced in a pressure tube and warmed in an oil bath at the corresponding temperature. Progress of the reactions was monitored by GC or TLC. Afterwards, the crude product was extracted using ethyl acetate (3x5 mL). The organic layer was dried and evaporated and further purified by column chromatography. The colloidal aqueous suspension was reused in other identical reactions (first cycle 83%; second cycle 84%). The final compounds **7a**–**f** were isolated and purified by flash chromatography (silica gel) using mixtures of *n*-hexane/EtOAc as eluent. They offered matching spectroscopic data with the analogous ones obtained by our group [23].

*(E)-n-Butyl 3-(4-methoxyphenyl)acrylate* (**7a**) [23]: Isolated 97 mg (83%, Table 2, entry 1op), 92 mg (79%, Table 2, entry 17) as colorless powder. M.p. 84–85 °C (*n*-hexane/EtOAc), Lit. 84–85 °C (pentanes/EtOAc) [44]. *R*_f_ = 0.3 (*n*-hexane). ^1^H NMR (400 MHz) δ (ppm): 7.58 (d, *J* = 16.0 Hz, 1H,), 7.39 (d, *J* = 4.2 Hz, 3H), 6.24 (d, *J* = 16.0 Hz, 1H), 4.13 (m, *J* = 7.0 Hz, 2H), 3.72 (s, 3H), 1.60–1.62 (m, 2H), 1.35–1.40 (m, 2H), 0.91 (t, *J* =7.0 Hz 3H). ^13^C NMR (100 MHz) δ (ppm) = 167.1, 161.2, 144.0, 129.5, 127.0, 115.5, 114.1, 64.0, 55.0, 30.7, 19.1, 13.6.
*(E)-4-Methoxystylbene* (**7b**) [23]: Isolated 84 mg (80%, Table 2, entry 10), 84 mg (80%, Table 2, entry 18) as colorless powder. M.p. 138–139 °C (*n*-hexane/EtOAc), Lit. 138 °C (water) [45]. *R*_f_ = 0.2 (*n*-hexane/EtOAc, 3:1). ^1^H NMR (400 MHz) δ (ppm): 7.46–7.52 (m, 3H), 7.36 (t, *J* = 7.6 Hz, 2H), 7.09 (d, *J* = 16.0 Hz, 2H), 6.99 (d, *J* = 16.4 Hz, 2H), 6.92 (d, *J* = 8.4 Hz, 2H), 3.89 (s, 3H). ^13^C NMR (100 MHz) δ (ppm): 159.3, 137.7, 130.1, 128.7, 128.2, 127.8, 127.2, 126.6, 126.3, 114.1, 55.3.
*(E)-n-Butyl cinnamate* (**7c**) [23]: Isolated 83 mg (81%, Table 2, entry 11), 78 mg (76%, Table 2, entry 15), 51 mg (50%, Table 2, entry 19), as colorless oil. *R*_f_ = 0.4 (*n*-hexane). ^1^H NMR (400 MHz) δ (ppm): 7.60 (d, *J* = 16.4 Hz, 1H), 7.43–7.45 (m, 2H), 7.29–7.30 (m, 3H), 6.36 (d, *J* = 16.0 Hz, 1H), 4.13 (t, *J* = 6.8 Hz, 2H), 1.59–1.62 (m, 2H), 1.34–1.36 (m, 2H), 0.88 (t, *J* = 7.6 Hz, 3H). ^13^C NMR (100 MHz) δ (ppm): 166.8, 144.4, 134.4, 130.1, 128.8, 128.0, 118.2, 64.3, 30.8, 19.2, 13.7.
*(E)-Stylbene* (**7d**) [23]: Isolated 72 mg (80%, Table 2, entry 12), 68 mg (75%, Table 2, entry 16), 49 mg (54%, Table 2, entry 20, as colorless solid. M.p. 124–125 °C (*n*-hexane/EtOAc), Lit. 123–125 °C (Merck, commercially available). *R*_f_ = 0.9 (*n*-hexane). ^1^H NMR (400 MHz) δ (ppm): 7.58 (d, *J* = 7.2 Hz, 4H), 7.42 (t, *J* = 7.0 Hz, 4H), 7.33 (t, *J* = 6.8 Hz, 2H), 7.18 (s, 2H). ^13^C NMR (100 MHz) δ (ppm): 137.4, 128.8, 127.8, 126.7.
*(E)-n-Butyl 3-(4-chlorophenyl) acrylate* (**7e**) [23]: Isolated 96 mg (81%, Table 2, entry 13) as colorless solid. M.p. 35–36 °C (*n*-hexane/EtOAc), Lit. 35–36 °C [46]. *R*_f_ = 0.4 (*n*-hexane). ^1^H NMR (300 MHz) δ (ppm): 7.65 (d, *J* = 16.0 Hz, 1H), 7.47 (d, *J* = 8.5 Hz, 2H), 7.37 (d, *J* = 8.5 Hz, 2H), 6.43 (d, *J* = 16.0 Hz, 1H), 4.24 (t, *J* = 6.6 Hz, 2H), 1.40-1.52 (m, 2H), 1.67–1.76 (m, 2H), 0.99 (t, *J* = 7.3 Hz, 3H). ^13^C NMR (100 MHz) δ (ppm): 166.7, 143.0, 136.0, 132.9, 129.4, 129.1, 118.8, 64.4, 30.7, 19.2, 13.7.
*(E)-1-Chloro-4-styrylbenzene* (**7f**) [23]: Isolated 82 mg (77%, Table 2, entry 14) as colorless solid. M.p. 127–128 °C (*n*-hexane/EtOAc), Lit. 126–128 °C [47]. *R*_f_ = 0.8 (*n*-hexane). ^1^H NMR (400 MHz) δ (ppm): 7.50–7.52 (m, 2H), 7.43–7.46 (m, 2H), 7.28–7.39 (m, 5H), 7.07 (s, 2H). ^13^C NMR (75 MHz) δ (ppm): 137.0, 135.8, 133.2, 129.3, 128.8, 128.7, 127.9, 127.7, 127.4, 126.6 ppm.

### 3.5. General Experimental Procedure for Hiyama Cross-Coupling

Aryl halide (0.5 mmol), catalyst **1** (1.9 mg, 0.0015 mmol, 0.3 mol%), triethoxyphenylsilane (0.119 mL, 0.6 mmol), NaOH (40 mg, 1 equiv) water (1.5 mL) were introduced in a pressure tube and warmed in an oil bath at the corresponding temperature. Progress of the reactions was monitored by GC or TLC. Afterwards, the crude product was extracted using ethyl acetate (3 × 5 mL). The organic layer was dried and evaporated and further purified by column chromatography. The colloidal aqueous suspension was reused in other identical reactions (first cycle 81%; second cycle 80%). The final compounds **5a**–**g** were isolated and purified by flash chromatography (silica gel) using mixtures of *n*-hexane/EtOAc as eluent. They offered matching spectroscopic data with the analogous ones obtained by our group [23].

(**5a**): Isolated 75 mg (81%, Table 3, entry 1op), 69 mg (75%, Table 3, entry 13), 45 mg (49%, Table 3, entry 16).

(**5b**): Isolated 67 mg (80%, Table 3, entry 10), 42 mg (50%, Table 3, entry 17).

(**5c**): Isolated 70 mg (78%, Table 3, entry 11).

(**5d**): Isolated 69 mg (76%, Table 3, entry 14).

(**5e**): Isolated 75 mg (80%, Table 3, entry 12).

(**5f**): Isolated 74 mg (75%, Table 3, entry 15).

(**5g**): Isolated 50 mg (56%, Table 3, entry 18).

### 3.6. General Experimental Procedure for Buchwald-Hartwig Cross-Coupling

Aryl halide (0.5 mmol), aniline (0.047 mL, 0.5 mmol), KOBu^t^ (224 mg, 2.00 mmol), **1** (6.5 mg, 0.005 mmol, 1.0 mol%), water (1.5 mL) were introduced in a pressure tube and warmed in an oil bath at the corresponding temperature for 24 h. Progress of the reactions was monitored by GC or TLC. Afterwards, the crude product was extracted using ethyl acetate (3 × 5 mL). The organic layer was dried and evaporated and further purified by column chromatography. The colloidal aqueous suspension was reused in other identical reactions (first cycle 41%; second cycle 41%). The final compounds **10a**–**c** were isolated and purified by flash chromatography (silica gel) using mixtures of *n*-hexane/EtOAc as eluent. They offered matching spectroscopic data with the analogous ones obtained in the literature [24,25,26].

*4-Methoxy-N-phenylaniline* (**10a**) [48]: Isolated 41 mg (41%, Table 4, entry 1op), 35 mg (35%, Table 4, entry 9) as colorless solid. M.p. 105–106 °C (*n*-hexane/EtOAc), Lit. 105 °C (*n*-hexane/EtOAc) [48]. *R*_f_ = 0.3 (*n*-hexane:EtOAc, 3:1). ^1^H NMR (400 MHz) δ (ppm): 7.40 (d, *J* = 8.4 Hz, 2H), 7.25-7.10 (m, 5H) 6.90 (d, *J* = 8.4 Hz, 2H), 5.50 (s,1H), 3.81 (s, 3H). ^13^C NMR (100 MHz) δ (ppm): 153.4, 145.7, 135.5, 129.1, 123.4, 120.1, 115.1, 55.3.
*4-Methyl-N-phenylaniline* (**10b**) [49]: Isolated 41 mg (45%, Table 4, entry 7) as colorless solid. M.p. 90–91 °C (*n*-hexane/EtOAc), Lit. 90 °C (*n*-hexane/EtOAc) [49] *R*_f_ = 0.5 (*n*-hexane:EtOAc, 3:1). ^1^H NMR (300 MHz) δ (ppm): 7.26 (t, *J* = 7.2 Hz, 2H), 7.20–6.90 (m, 6H), 6.85 (m, 1H), 5.61 (s, 1H), 2.32 (s, 3H). 13 C-NMR (75 MHz) δ (ppm): 143.9, 140.2, 130.9, 129.8, 129.1, 120.3, 118.8, 116.9, 20.6.
*4-Chloro-N-phenylaniline* (**10c**) [49]: Isolated 59 mg (58%, Table 4, entry 8) as colorless solid. M.p. 68–69 °C (*n*-hexane/EtOAc), Lit. 69 °C (*n*-hexane/EtOAc) [49]. *R*_f_ = 0.3 (*n*-hexane:EtOAc, 3:1). ^1^H NMR (300 MHz) δ (ppm): 7.35–7.20 (m, 2H), 7.20 (dt, *J* = 9.0, 3.3 Hz, 2H), 7.70–6.90 (m, 5H), 5.60 (s, 1H). ^13^C-NMR (75 MHz) δ (ppm): 142.5, 141.9, 129.3, 129.2, 125.5, 121.0, 118.5, 118.2.

### 3.7. General Experimental Procedure for Hirao Cross-Coupling

Aryl halide (0.5 mmol), triethylphosphite (0.166 mL, 1.0 mmol), Et_3_N (0.342 mL, 2.5 mmol), **1** (3.3 mg, 0.0025 mmol, 0.5 mol%), water (1.5 mL) were introduced in a pressure tube and warmed in an oil bath at the corresponding temperature for 8 h. Progress of the reactions was monitored by GC or TLC. Afterwards, the crude product was extracted using ethyl acetate (3 × 5 mL). The organic layer was dried, evaporated and further purified by column chromatography. The colloidal aqueous suspension was reused in other identical reactions (six different catalytic cycles, see Figure 5b) or using for the characterization of the nanoparticles. The final compounds **12a**–**d** were isolated and purified by flash chromatography (silica gel) using mixtures of *n*-hexane/EtOAc as eluent. They offered matching spectroscopic data with the analogous ones obtained in the literature [23].

*Diethyl 4-methoxyphenylphosphonate* (**12a**) [23]: Isolated 107 mg (88%, Table 5, entry 1op), 88 mg (72%, Table 5, entry 14), 72 mg (59%, Table 5, entry 17) as colorless oil. *R*_f_ = 0.4 (*n*-hexane:EtOAc, 3:1). ^1^H NMR (300 MHz) δ (ppm): 8.30 (dd, *J* = 8.7 and 3.3 Hz, 1H), 8.00 (dd, *J* = 12.7 and 8.7 Hz, 1H), 4.27–4.06 (m, 4H), 1.34 (t, *J* = 6.9 Hz, 6H). ^13^C NMR (75 MHz) δ (ppm): 150.2 (d, *J* = 3.7 Hz), 135.8 (d, *J* = 185.2 Hz), 133.0 (d, *J* = 10.5 Hz), 123.3 (d, *J* = 15.0 Hz), 62.7 (d, *J* = 5.2 Hz), 16.3 (d, *J* = 6.0 Hz), 16.1 (d, *J* = 6.7 Hz).
*Diethyl phenylphosphonate* (**12b**) [23]: Isolated 97 mg (91%, Table 5, entry 10), 80 mg (75%, Table 5, entry 12), 73 mg (68%, Table 5, entry 15) as colorless oil. *R*_f_ = 0.5 (*n*-hexane:EtOAc, 3:1). ^1^H NMR (300 MHz) δ (ppm): 7.80 (dd, *J* = 13.2 and 8.4 Hz, 2H), 7.50–7.48 (m, 1H), 7.48–7.40 (m, 2H), 4.15–4.05 (m, 4H), 1.30 (t, *J* = 6.8 Hz, 6H). ^13^C NMR (75 MHz) δ (ppm): 132.3 (d, *J* = 3.0 Hz), 131.7 (d, *J* = 10.0 Hz), 128.4 (d, *J* = 15.0 Hz), 128.3 (d, *J* = 186.0 Hz), 62.0 (d, *J* = 5.0 Hz), 16.3 (d, *J* = 7.0 Hz).
*Diethyl 4-chlorophenylphosphonate* (**12c**) [23]: Isolated 105 mg (85%, Table 5, entry 11) as colorless oil. *R*_f_ = 0.3 (*n*-hexane:EtOAc, 3:1). ^1^H NMR (400 MHz) δ (ppm): 7.76 (dd, *J* = 12.8 and 8.4 Hz, 2H), 7.46 (dd, *J* = 8.2 and 3.6 Hz, 2H), 4.20-4.05 (m, 4H), 1.34 (t, *J* = 7.2 Hz, 6H). ^13^C NMR (100 MHz) δ (ppm): 139.9 (d, *J* = 4.0 Hz), 133.2 (d, *J* = 10.0 Hz), 128.8 (d, *J* = 16.0 Hz), 126.9 (d, *J* = 190.0 Hz), 62.2 (d, *J* = 5.0 Hz), 16.3 (d, *J* = 7.0 Hz).
*Diethyl 4-methylphenylphosphonate* (**12d**) [23]: Isolated 82 mg (72%, Table 5, entry 13), 75 mg (66%, Table 5, entry 16) as colorless oil. *R*_f_ = 0.6 (*n*-hexane:EtOAc, 3:1). ^1^H NMR (400 MHz) δ (ppm): 7.72 (dd, *J* = 13.2 and 8.1 Hz, 2H), 7.29 (dd, *J* = 8.1 and 3.3 Hz, 2H), 4.21–4.01 (m, 4H), 2.42 (s, 3H), 1.33 (t, *J* = 6.9 Hz, 6H). ^13^C NMR (75 MHz) δ (ppm): 142.9 (d, *J* = 3.0 Hz), 131.8 (d, *J* = 9.7 Hz), 129.2 (d, *J* = 15.0 Hz), 124.9 (d, *J* = 188.2 Hz), 61.9 (d, *J* = 5.2 Hz), 21.6 (d, *J* = 6.7 Hz), 16.1 (d, *J* = 6.7 Hz).

### 3.8. General Experimental Procedure for Sonogashira-Hagihara Cross-Coupling

Aryl halide (0.5 mmol), terminal alkyne (0.55 mmol), DABCO (84 mg, 0.75 mmol), **1** (3.3 mg, 0.0025 mmol, 0.5 mol%), water (1.5 mL) were introduced in a pressure tube and warmed in an oil bath at the corresponding temperature for 10 h. Progress of the reactions was monitored by GC or TLC. Afterwards, the crude product was extracted using ethyl acetate (3 × 5 mL). The organic layer was dried, evaporated and further purified by column chromatography. The colloidal aqueous suspension was reused in other identical reactions (first cycle 91%; second cycle 92%). Final compounds **14a**–**f** were isolated and purified by flash chromatography (silica gel) using mixtures of *n*-hexane/EtOAc as eluent. They offered matching spectroscopic data with the analogous ones obtained in the literature [28].

*1-Methoxy-4-(phenylethynyl)-benzene* (**14a**) [50]: Isolated 95 mg (91%, Table 6, entry 1op), 75 mg (72%, Table 6, entry 16), 63 mg (61%, Table 6, entry 20) as colorless crystals. M.p. 68–69 °C (*n*-hexane/EtOAc), Lit. 65 °C (*n*-hexane/EtOAc) [50]. *R*_f_ = 0.8 (*n*-hexane:EtOAc, 3:1). ^1^H NMR (300 MHz) δ (ppm): 7.56–7.48 (m, 4H), 7.36–7.33 (m, 3H), 6.80 (dd, *J* = 4.2 and 2.0 Hz, 1H,), 3.83 (s, 3H). ^13^C NMR (75 MHz) δ (ppm) 159.2, 133.0, 131.4, 128.3, 127.9, 123.5, 115.2, 113.9, 89.3, 88.0, 55.2.
*1,2-Diphenylethyne* (**14b**) [50]: Isolated 77 mg (87%, Table 6, entry 10), 67 mg (75%, Table 6, entry 15), 61 mg (68%, Table 6, entry 19) as colorless crystals. M.p. 59–60 °C (*n*-hexane/EtOAc), Lit. 59–60 °C (*n*-hexane) [50]. *R*_f_ = 0.5 (*n*-hexane). ^1^H NMR (300 MHz) δ (ppm): 7.54–7.51 (m, 4H), 7.36–7.31 (m, 6H). ^13^C NMR (75 MHz) δ (ppm) 131.6, 128.3, 128.2, 123.2, 89.3.
*1-Chloro-4-(phenylethynyl)benzene* (**14c**) [50]: Isolated 90 mg (85%, Table 6, entry 11) as colorless crystals. M.p. 85–87 °C (*n*-hexane/EtOAc), Lit. 84 °C (*n*-hexane) [50]. *R*_f_ = 0.9 (*n*-hexane/EtOAc, 3:1). ^1^H NMR (300 MHz) δ (ppm): 7.55–7.48 (m, 2H), 7.41–7.47 (m, 2H), 7.37–7.27 (m, 5H). ^13^C NMR (75 MHz) δ (ppm) 134.2, 132.8, 131.6, 128.7, 128.45, 128.36, 122.9, 121.8, 90.3, 88.2.
*4-(Phenylethynyl)benzonitrile* (**14d**) [28,50]: Isolated 84 mg (83%, Table 6, entry 12) as colorless crystals. M.p. 105–107 °C (*n*-hexane/EtOAc), Lit. 106–108 °C (*n*-hexane/EtOAc) [51]. *R*_f_ = 0.8 (*n*-hexane/EtOAc, 3:1). ^1^H NMR (400 MHz) δ (ppm): 7.68–7.61 (m, 4H), 7.58–7.54 (m, 2H), 7.42–7.39 (m, 3H). ^13^C NMR (100 MHz) δ (ppm): 132.0, 132.0, 131.8, 129.1, 128.5, 128.2, 122.2, 118.5, 111.4, 93.7, 87.7).
*3-Phenyl-2-propyn-1-ol* (**14e**) [52]: Isolated 53 mg (80%, Table 6, entry 13), 46 mg (70%, Table 6, entry 17), 46 mg (69%, Table 6, entry 21) as colorless solid. M.p 120–121 °C (*n*-hexane/EtOAc), Lit. 119–121 °C [52]. *R*_f_ = 0.2 (*n*-hexane/EtOAc, 3:1). ^1^H NMR (300MHz) δ (ppm): 7.40–7.45 (m, 2H), 7.30–7.35 (m, 3H), 4.5 (s, 2H). ^13^C NMR (75 MHz) δ (ppm): 131.7, 128.5, 128.3, 122.3, 87.1, 85.6, 51.6.
*3-(4-Methoxyphenyl)-2-propyn-1-ol* (**14f**) [53]: Isolated 65 mg (80%, Table 6, entry 14), 56 mg (69%, Table 6, entry 18), 57 mg (70%, Table 6, entry 22) as colorless solid. M.p. 65–66 °C (*n*-hexane/EtOAc), Lit. 65–68 °C [53]. *R*_f_ = 0.3 (*n*-hexane/EtOAc, 3:1). ^1^H NMR (300MHz) δ (ppm): 7.35 (d, *J* = 8.9 Hz, 2H), 6.83 (d, *J* = 8.9 Hz, 2H), 4.46 (s, 2H), 3.80 (s, 3H). ^13^C NMR (75 MHz) δ (ppm): 159.6, 133.4, 114.4, 114.0, 85.9, 85.4, 55.6, 51.8.

### 3.9. General Experimental Procedure for the Recycling Tests in (a) Suzuki-Miyaura and (b) Hirao Cross-Couplings

(a) Measures of 4-Iodoanisole (117 mg, 0.5 mmol), phenylboronic acid (92 mg, 0.75 mmol), K_2_CO_3_ (103 mg, 0.75 mmol), complex **1** (1.3 mg, 0.001 mmol, 0.2 mol%) and water (1.5 mL) were introduced in a pressure tube and warmed in an oil bath at the corresponding temperature for 24 h. Afterwards, crude product was extracted using ethyl acetate (3 × 5 mL). The organic layer was separated, and the aqueous suspension of nanoparticles was mixed with 4-iodoanisole (117 mg, 0.5 mmol), phenylboronic acid (92 mg, 0.75 mmol), and K_2_CO_3_ (103 mg, 0.75 mmol), and the process was repeated as described before.
(b) Measures of 4-Iodoanisole (117 mg, 0.5 mmol), triethylphosphite (0.166 mL, 1.0 mmol), Et_3_N (0.342 mL, 2.5 mmol), **1** (3.3 mg, 0.0025 mmol, 0.5 mol%), water (1.5 mL) were introduced in a pressure tube and warmed in an oil bath at the corresponding temperature for 8 h. Afterwards, crude product was extracted using ethyl acetate (3 × 5 mL). The organic layer was separated, and the aqueous suspension of nanoparticles was mixed with 4-iodoanisole (117 mg, 0.5 mmol), triethylphosphite (0.166 mL, 1.0 mmol), and Et_3_N (0.342 mL, 2.5 mmol), and the process repeated as described before.

## 4. Conclusions

In this work, a suspension of nanoparticles in water (generated from complex **1**) was introduced as an effective and versatile catalyst in six classical cross-coupling reactions. The tolerance to multiple functional groups was also demonstrated in the Suzuki–Miyaura, Mizoroki–Heck, Hiyama, Buchwald–Hartwig, Hirao and Sonogashira–Hagihara reactions. The less productive transformation was Buchwald–Hartwig coupling, affording modest chemical yields. The reaction with iodides and bromides was satisfactory, whilst aryl chlorides required harsh conditions. Temperature is also a key parameter for performing chemoselective transformations; thus, at lower temperatures, the carbon–iodine bond was activated in the presence of a carbon–chlorine bond. The nanoparticles were characterized, and their presence was confirmed in the coupling reactions. The catalyst loading was very small, and the suspension in water was easily separated and reused for up to six catalytic batches, maintaining the chemical yield. The degree of sinterization was very low after this sixth stage. The processes can be classified as heterogeneous, sustainable and able to take care of the environment using the lower amount of waste chemicals and solvents. Water ensures a fast and homogeneous dispersion of nanoparticles and favors the approach of the organic reagents, avoiding the solvation of them, as is what occurred in the reactions performed with other organic solvents (see Table 1, Table 2, Table 3, Table 4, Table 5 and Table 6). It was also demonstrated that there was already-known lower reactivity of aryl chlorides due to their higher dissociation energies. Concerning the efficiency of this process *versus* catalysis conducted in the absence of nanoparticles, it is remarkable that there was a higher catalytic surface of nanoparticles, and also that there was higher stability. This last fact was demonstrated by the recyclability study performed in Suzuki–Miyaura and Hirao cross-coupling reactions.

## Figures and Tables

**Table 1:** Optimization and scope of the Suzuki–Miyaura coupling using complexes **1** or **2** ^1^.

Entry	Pd Source	Ar ^1^-Hal 3	Ar ^2^ 4	Solvent	T (°C)	5	Yield (%) ^2^
1op	**1**	4-MeO-C_6_H_4_I	Ph	H_2_O	90	**5a**	89
2op	**2**	4-MeO-C_6_H_4_I	Ph	H_2_O	90	**5a**	65
3op	**1**	4-MeO-C_6_H_4_I	Ph	PhMe	90	**5a**	37
4op	**1**	4-MeO-C_6_H_4_I	Ph	1,4-dioxane	90	**5a**	88
5op	**1**	4-MeO-C_6_H_4_I	Ph	DMF	90	**5a**	43
6op ^3^	**1**	4-MeO-C_6_H_4_I	Ph	H_2_O	90	**5a**	66
7op	**1**	4-MeO-C_6_H_4_I	Ph	H_2_O	70	**5a**	78
8	**1**	4-MeC_6_H_4_I	Ph	H_2_O	90	**5b**	89
9	**1**	3-(CN)C_6_H_4_I	Ph	H_2_O	90	**5c**	88
10	**1**	3-(CHO)C_6_H_4_I	Ph	H_2_O	90	**5d**	90
11	**1**	4-ClC_6_H_4_I	Ph	H_2_O	90	**5e**	90
12	**1**	PhI	4-MeO-C_6_H_4_	H_2_O	90	**5a**	87
13	**1**	PhI	4-Me-C_6_H_4_	H_2_O	90	**5b**	87
14	**1**	4-MeO-C_6_H_4_Br	Ph	H_2_O	110	**5a**	79
15	**1**	3-(CHO)C_6_H_4_Br	Ph	H_2_O	110	**5d**	81
16	**1**	4-(Ac)C_6_H_4_Br	Ph	H_2_O	110	**5f**	80
17	**1**	4-MeO-C_6_H_4_Cl	Ph	H_2_O	150	**5a**	54
18	**1**	4-Me-C_6_H_4_Cl	Ph	H_2_O	150	**5b**	68
19	**1**	4-(CN)-C_6_H_4_Cl	Ph	H_2_O	150	**5g**	70

op = optimization test. ^1^ Typical procedure: 4-iodoanisole (0.5 mmol), phenylboronic acid (0.75 mmol), K_2_CO_3_ (0.75 mmol), **1** (0.2 mol%), water (1.5 mL) were introduced in a pressure tube and warmed in an oil bath at the corresponding temperature for 24 h. ^2^ Chemical yields isolated after flash chromatography. ^3^ A measure of 0.1 mol% of palladium complex **1** was employed.

**Table 2:** Optimization and scope of the Mizoroki–Heck reaction using complexes **1** or **2** ^1^.

Entry	Pd Source	Ar ^1^-Hal 3	R 6	Solvent	T (°C)	7	Yield (%) ^2^
1op	**1**	4-MeO-C_6_H_4_I	CO_2_Bu^n^	H_2_O	100	**7a**	83
2op	**2**	4-MeO-C_6_H_4_I	CO_2_Bu^n^	H_2_O	100	**7a**	64
3op	**1**	4-MeO-C_6_H_4_I	CO_2_Bu^n^	PhMe	100	**7a**	80
4op	**1**	4-MeO-C_6_H_4_I	CO_2_Bu^n^	1,4-dioxane	100	**7a**	77
5op	**1**	4-MeO-C_6_H_4_I	CO_2_Bu^n^	DMF	100	**7a**	80
6op ^3^	**1**	4-MeO-C_6_H_4_I	CO_2_Bu^n^	H_2_O	100	**7a**	81
7op ^4^	**1**	4-MeO-C_6_H_4_I	CO_2_Bu^n^	H_2_O	100	**7a**	58
8op ^5^	**1**	4-MeO-C_6_H_4_I	CO_2_Bu^n^	H_2_O	100	**7a**	43
9op ^6^	**1**	4-MeO-C_6_H_4_I	CO_2_Bu^n^	H_2_O	90	**7a**	66
10	**1**	4-MeO-C_6_H_4_I	Ph	H_2_O	100	**7b**	80
11	**1**	PhI	CO_2_Bu^n^	H_2_O	100	**7c**	81
12	**1**	PhI	Ph	H_2_O	100	**7d**	80
13	**1**	4-Cl-C_6_H_4_I	CO_2_Bu^n^	H_2_O	100	**7e**	81
14	**1**	4-Cl-C_6_H_4_I	Ph	H_2_O	100	**7f**	77
15	**1**	PhBr	CO_2_Bu^n^	H_2_O	120	**7c**	76
16	**1**	PhBr	Ph	H_2_O	120	**7d**	75
17	**1**	4-MeO-C_6_H_4_Br	CO_2_Bu^n^	H_2_O	120	**7a**	79
18	**1**	4-MeO-C_6_H_4_Br	Ph	H_2_O	120	**7b**	80
19	**1**	PhCl	CO_2_Bu^n^	H_2_O	130 ^6^	**7c**	50
20	**1**	PhCl	Ph	H_2_O	130 ^6^	**7d**	54

op = optimization test. ^1^ Typical procedure: 4-iodoanisole (0.5 mmol), olefin (0.55 mmol), triethylamine (1 mmol), H_2_O (1.5 mL) and **1** (0.3 mol%), were introduced in a pressure tube and warmed in an oil bath at the corresponding temperature. ^2^ Chemical yields isolated after flash chromatography. ^3^ K_2_CO_3_ was used as base. ^4^ NaOH was used as base. ^5^ A measure of 0.2 mol% of palladium complex **1** was employed. ^6^ Represents 24 h of reaction.

**Table 3:** Optimization and scope of the Hiyama cross-coupling using complexes **1** or **2** ^1^.

Entry	Pd Source	Ar ^1^-Hal 3	Solvent	T (°C)	5	Yield (%) ^2^
1op	**1**	4-MeO-C_6_H_4_I	H_2_O	100	**5a**	81
2op	**2**	4-MeO-C_6_H_4_I	H_2_O	100	**5a**	55
3op	**1**	4-MeO-C_6_H_4_I	PhMe	100	**5a**	80
4op	**1**	4-MeO-C_6_H_4_I	1,4-dioxane	100	**5a**	80
5op	**1**	4-MeO-C_6_H_4_I	DMF	100	**5a**	81
6op ^3^	**1**	4-MeO-C_6_H_4_I	H_2_O	100	**5a**	81
7op ^4^	**1**	4-MeO-C_6_H_4_I	H_2_O	100	**5a**	58
8op	**1**	4-MeO-C_6_H_4_I	H_2_O	90	**5a**	53
9op ^5^	**1**	4-MeO-C_6_H_4_I	H_2_O	100	**5a**	66
10	**1**	4-Me-C_6_H_4_I	H_2_O	100	**5b**	80
11	**1**	3-(CN)-C_6_H_4_I	H_2_O	100	**5c**	78
12	**1**	4-Cl-C_6_H_4_I	H_2_O	100	**5e**	80
13	**1**	4-MeO-C_6_H_4_Br	H_2_O	110	**5a**	75
14	**1**	3-(CHO)-C_6_H_4_Br	H_2_O	110	**5d**	76
15	**1**	4-(Ac)-C_6_H_4_Br	H_2_O	110	**5f**	75
16	**1**	4-MeO-C_6_H_4_Cl	H_2_O	120 ^6^	**5a**	49
17	**1**	4-Me-C_6_H_4_Cl	H_2_O	120 ^6^	**5b**	50
18	**1**	4-(CN)-C_6_H_4_Cl	H_2_O	120 ^6^	**5g**	56

op = optimization test. ^1^ Typical procedure: 4-iodoanisole (0.5 mmol), catalyst **1** (0.3 mol%), triethoxyphenylsilane (0.6 mmol), NaOH (0.5 mmol) water (1.5 mL) were introduced in a pressure tube and warmed in an oil bath at the corresponding temperature. ^2^ Chemical yields isolated after flash chromatography. ^3^ K_2_CO_3_ was used as base. ^4^ Et_3_N was used as base. ^5^ A measure of 0.2 mol% of palladium complex **1** was employed. ^6^ Represents 24 h of reaction.

**Table 4:** Optimization and scope of Buchwald-Hartwig reaction using complexes **1** or **2** ^1^.

Entry	Pd Source	Ar ^1^-Hal 3	T (°C)	10	Yield (%) ^2^
1op	**1**	4-MeO-C_6_H_4_I	100	**10a**	41
2op	**2**	4-MeO-C_6_H_4_I	100	**10a**	<10
3op	**1**	4-MeO-C_6_H_4_I	120	**10a**	40
4op ^3^	**1**	4-MeO-C_6_H_4_I	100	**10a**	38
5op	**1**	4-MeO-C_6_H_4_I	90	**10a**	nr
6op ^4^	**1**	4-MeO-C_6_H_4_I	100	**10a**	38
7	**1**	4-Me-C_6_H_4_I	100	**10b**	45
8	**1**	4-Cl-C_6_H_4_I	100	**10c**	58
9	**1**	4-MeO-C_6_H_4_Br	120	**10a**	35

op = optimization test. ^1^ Typical procedure: 4-iodoanisole (0.5 mmol), aniline (0.5 mmol), KOBu^t^ (2.00 mmol), **1** (1.0 mol%), water (1.5 mL) were introduced in a pressure tube and warmed in an oil bath at the corresponding temperature for 24 h. ^2^ Chemical yields isolated after flash chromatography. ^3^ K_3_PO_4_ was used as base. ^4^ A measure of 2.0 mol% of palladium complex **1** was employed.

**Table 5:** Optimization and scope of Hirao reaction using complexes **1** or **2** ^1^.

Entry	Pd Source	Ar ^1^-Hal 3	Solvent	T (°C)	12	Yield (%) ^2^
1op	**1**	4-MeO-C_6_H_4_I	H_2_O	100	**12a**	88
2op	**2**	4-MeO-C_6_H_4_I	H_2_O	100	**12a**	51
3op	**1**	4-MeO-C_6_H_4_I	PhMe	100	**12a**	75
4op	**1**	4-MeO-C_6_H_4_I	1,4-dioxane	100	**12a**	76
5op	**1**	4-MeO-C_6_H_4_I	DMF	100	**12a**	74
6op ^3^	**1**	4-MeO-C_6_H_4_I	H_2_O	100	**12a**	42
7op ^4^	**1**	4-MeO-C_6_H_4_I	H_2_O	100	**12a**	58
8op	**1**	4-MeO-C_6_H_4_I	H_2_O	90	**12a**	76
9op ^5^	**1**	4-MeO-C_6_H_4_I	H_2_O	100	**12a**	38
10	**1**	PhI	H_2_O	100	**12b**	91
11	**1**	4-Cl-C_6_H_4_I	H_2_O	100	**12c**	85
12	**1**	PhBr	H_2_O	100	**12b**	75
13	**1**	4-Me-C_6_H_4_Br	H_2_O	100	**12d**	72
14	**1**	4-MeO-C_6_H_4_Br	H_2_O	100	**12a**	72
15	**1**	PhCl	H_2_O	120 ^6^	**12b**	68
16	**1**	4-Me-C_6_H_4_Cl	H_2_O	120 ^6^	**12d**	66
17	**1**	4-MeO-C_6_H_4_Cl	H_2_O	120 ^6^	**12a**	59

op = optimization test. ^1^ Typical procedure: 4-iodoanisole (0.5 mmol), triethylphosphite (1.0 mmol), Et_3_N (2.5 mmol), **1** (0.5 mol%), water (1.5 mL) were introduced in a pressure tube and warmed in an oil bath at the corresponding temperature for 8 h. ^2^ Chemical yields isolated after flash chromatography. ^3^ NaOH was used as base. ^4^ K_2_CO_3_ was used as base. ^5^ A measure of 0.3 mol% of palladium complex **1** was employed. ^6^ Represents 24 h of reaction.

**Table 6:** Optimization and scope of Sonogashira–Hagihara cross-coupling using complexes **1** or **2** ^1^.

Entry	Pd Source	Ar ^1^-Hal 3	R 13	Solvent	T (°C)	14	Yield (%) ^2^
1op	**1**	4-MeO-C_6_H_4_I	Ph	H_2_O	90	**14a**	91
2op	**2**	4-MeO-C_6_H_4_I	Ph	H_2_O	90	**14a**	81
3op	**1**	4-MeO-C_6_H_4_I	Ph	PhMe	90	**14a**	85
4op	**1**	4-MeO-C_6_H_4_I	Ph	1,4-dioxane	90	**14a**	90
5op	**1**	4-MeO-C_6_H_4_I	Ph	DMF	90	**14a**	90
6op ^3^	**1**	4-MeO-C_6_H_4_I	Ph	H_2_O	90	**14a**	72
7op ^4^	**1**	4-MeO-C_6_H_4_I	Ph	H_2_O	90	**14a**	50
8op	**1**	4-MeO-C_6_H_4_I	Ph	H_2_O	70	**14a**	46
9op ^5^	**1**	4-MeO-C_6_H_4_I	Ph	H_2_O	90	**14a**	29
10	**1**	PhI	Ph	H_2_O	90	**14b**	87
11	**1**	4-Cl-C_6_H_4_I	Ph	H_2_O	90	**14c**	85
12	**1**	4-(CN)-C_6_H_4_I	Ph	H_2_O	90	**14d**	83
13	**1**	PhI	CH_2_OH	H_2_O	90	**14e**	80
14	**1**	4-MeO-C_6_H_4_I	CH_2_OH	H_2_O	90	**14f**	80
15	**1**	PhBr	Ph	H_2_O	90	**14b**	75
16	**1**	4-MeO-C_6_H_4_Br	Ph	H_2_O	90	**14a**	72
17	**1**	PhBr	CH_2_OH	H_2_O	90	**14e**	70
18	**1**	4-MeO-C_6_H_4_Br	CH_2_OH	H_2_O	90	**14f**	69
19	**1**	PhCl	Ph	H_2_O	120 ^6^	**14b**	68
20	**1**	4-MeO-C_6_H_4_Cl	Ph	H_2_O	120 ^6^	**14a**	61
21	**1**	PhCl	CH_2_OH	H_2_O	120 ^6^	**14e**	69
22	**1**	4-MeO-C_6_H_4_Cl	CH_2_OH	H_2_O	120 ^6^	**14f**	70

Op = optimization test. ^1^ Typical procedure: 4-iodoanisole (0.5 mmol), phenylacetylene (0.55 mmol), DABCO (0.75 mmol), **1** (0.5 mol%), water (1.5 mL) were introduced in a pressure tube and warmed in an oil bath at the corresponding temperature for 10 h. ^2^ Chemical yields isolated after flash chromatography. ^3^ Et_3_N was used as base. ^4^ K_2_CO_3_ was used as base. ^5^ A measure of 0.3 mol% of palladium complex **1** was employed. ^6^ Represents 24 h of reaction.

**Table 7:** Average of the elemental composition of the three samples of nanoparticles after a Suzuki–Miyaura reaction and other three samples after Hirao cross-coupling.

Analysis	C (%)	H (%)	N (%)	O (%)	S (%)	Pd (%)
XPS	0.6–0.8	nd	0.2–0.3	0.0–0.1	0.2–0.3	98.9–98.3
ICP	0.7–0.8	nd	nd	nd	0.2–0.3	98.9–98.5
EA	0.7–0.8	0.2–0.2	0.2–0.3	nd	0.2–0.2	nd

nd = not determined.

## Data Availability

Data are contained within the article and Supplementary Materials.

## Supplementary Materials

The following supporting information can be downloaded at: https://www.mdpi.com/article/10.3390/molecules29051138/s1, NMR spectra.
