# Supplementary material for: Stabilized Palladium Nanoparticles from Bis-(N-benzoylthiourea) Derived-PdII Complexes as Efficient Catalysts for Sustainable Cross-Coupling Reactions in Water
Source: Molecules. 2024 Mar 4;29(5):1138. doi: 10.3390/molecules29051138 (PMC10933781; doi:10.3390/molecules29051138)
Supplement: Supplementary file 1 [file molecules-29-01138-s001.zip › molecules-2874651-supplementary.pdf]

Stabilized Palladium Nanoparticles from Bis-(*N*-benzoylthiourea) Derived-Pd^{II} Complexes as Efficient Catalysts for Sustainable Cross-Coupling Reactions in Water

Samet Poyraz [1], H. Ali Döndaş [1,2,*], Samet Belveren [1], Senanur Taş [2], Raquel Hidalgo-León [3], José Trujillo-Sierra [3], Lesly V. Rodríguez-Flórez [3], Mª de Gracia Retamosa [3], Ana Sirvent [3], Mohammad Gholinejad [4,5], Sara Sobhani [6] and José M. Sansano [3,*]

1 Department of Basic Pharmaceutical Sciences, Faculty of Pharmacy, Çukurova University, Balcalı, Adana 01330, Türkiye
2 Department of Biotechnology, Institute of Natural and Applied Sciences, Çukurova University, Balcalı, Adana 01330, Türkiye
3 Departamento de Química Orgánica, Instituto de Síntesis Orgánica (ISO), Centro de Innovación en Química Avanzada (ORFEO-CINQA), University of Alicante, P.O. Box 99, Alicante 03690, Spain
4 Department of Chemistry, Institute for Advanced Studies in Basic Sciences (IASBS), P.O. Box 45195-1159, Zanjan 45137-66731, Iran
5 Research Center for Basic Sciences & Modern Technologies (RBST), Institute for Advanced Studies in Basic Sciences (IASBS), Zanjan 45137-66731, Iran
6 Department of Chemistry, College of Sciences, Shiraz University, Shiraz 71454, Iran
* Correspondence: adondas@cu.edu.tr (H.A.D.); jmsansano@ua.es (J.M.S.)

Complex 2

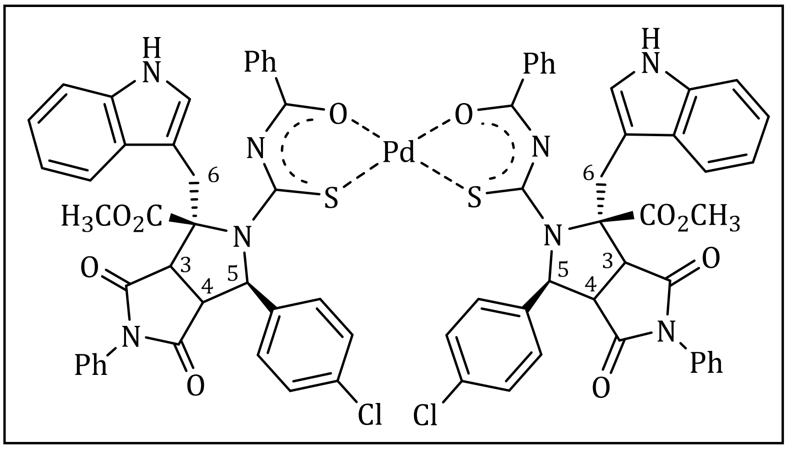

Brownish-yellow solid, 123 mg, 90% yield; mp 253–255 °C (MeOH, decomp.); IR (cm^{-1}) ν_{max}: 3417, 3060, 2952, 1790, 1716, 1495, 1391, 1258, 1201, 1168, 1093, 743. δ_H (400 MHz, $CDCl_3$): 8.39 (s, 2H, N-*H*), 8.20 (d, 4H, *J* = 7.3 Hz, Ar-*H*), 7.73 (d, 2H, *J* = 7.8 Hz, Ar-*H*), 7.55 (d, 2H, *J* = 7.3 Hz, Ar-*H*), 7.51-7.36 (m, 9H, Ar-*H*), 7.32-7.26 (m, 9H, Ar-*H*), 7.19 (d, 4H, *J* = 7.7 Hz, Ar-*H*), 7.10 (d, 2H, *J* = 1.9 Hz, Ar-*H*), 6.52-6.45 (m, 4H, Ar-*H*), 5.40 (d, 2H, *J* = 11.0 Hz, 5-*H*), 4.52 (d, 2H, *J* = 15.10 Hz, 6-*H*),

3.95 (s, 6H, OCH_3), 3.89 (d, 2H, J = 15.00 Hz, 6'-H), 3.84 (d, 2H, J = 9.5 Hz, 3-H), 2.60 (dd, 2H, J = 10.8 Hz, 9.1 Hz, 4-H). δ_C (100 MHz, $CDCl_3$): 172.66 (2xC=S), 172.63 (2xC=O), 172.25 (2xC=O), 172.00 (2xC=O), 169.67 (2xC=O), 136.02 (2C), 135.91 (2C), 135.83 (2C), 133.88 (2C), 132.59 (2C), 130.74 (2C), 130.12 (6C), 129.03 (6C), 128.74 (2C), 128.34 (6C), 127.75 (2C), 125.72 (6C), 124.33 (2C), 122.89 (2C), 120.78 (2C), 117.85 (2C), 111.96 (2C), 108.72 (2C), 68.90 (2C), 54.13 (2C), 53.15 (2C), 48.59 (2C), 31.77 (2C), 30.89 (2C). HRMS: calculated for $C_{74}H_{60}Cl_2N_8O_{10}PdS_2$: 1462.7717, found: 1462.7711. EA calculated for $C_{74}H_{60}Cl_2N_8O_{10}PdS_2$: C 60.8, H 4.1, N 7.7, S 4.4 %; found: C 60.5, H 4.4, N 7.7, S 4.5 %

^{1}H NMR spectra of complex 2

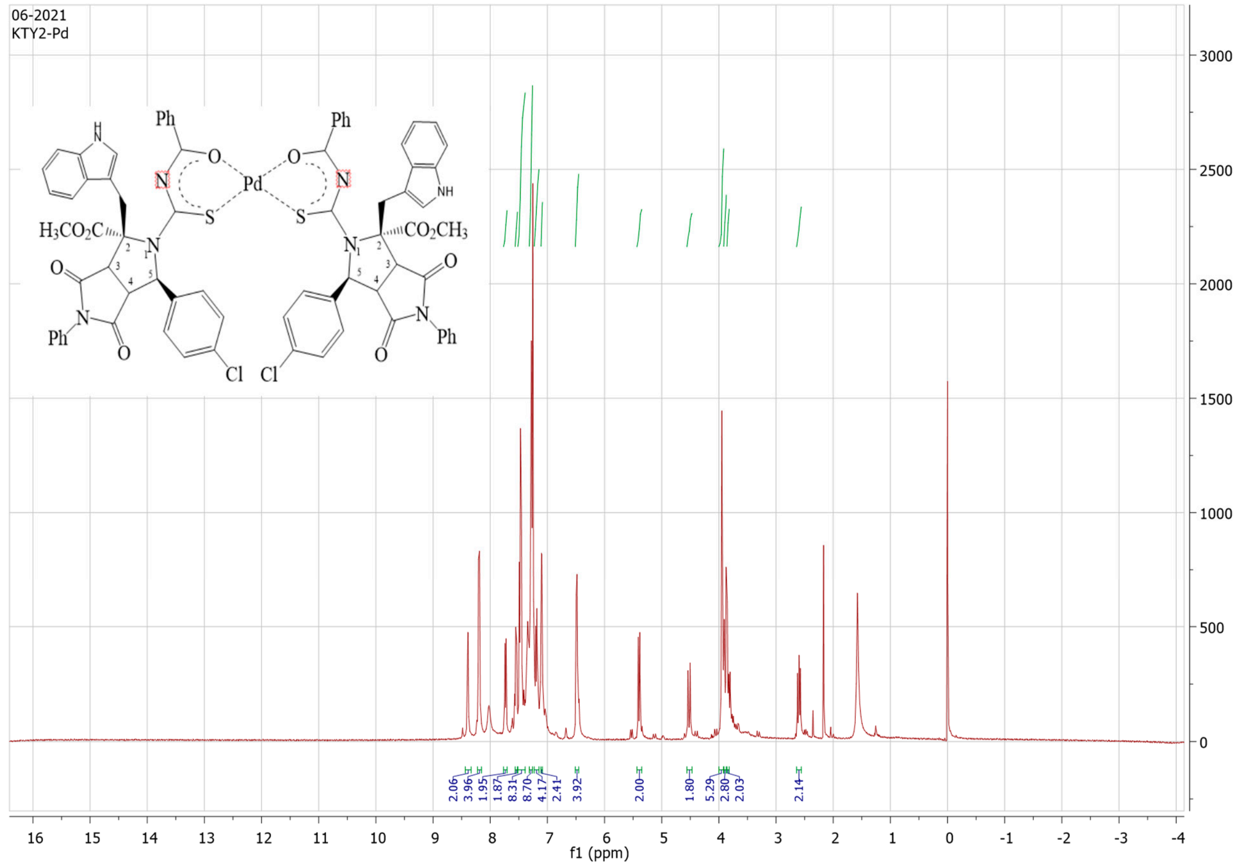

^{13}C-NMR spectra of complex 2

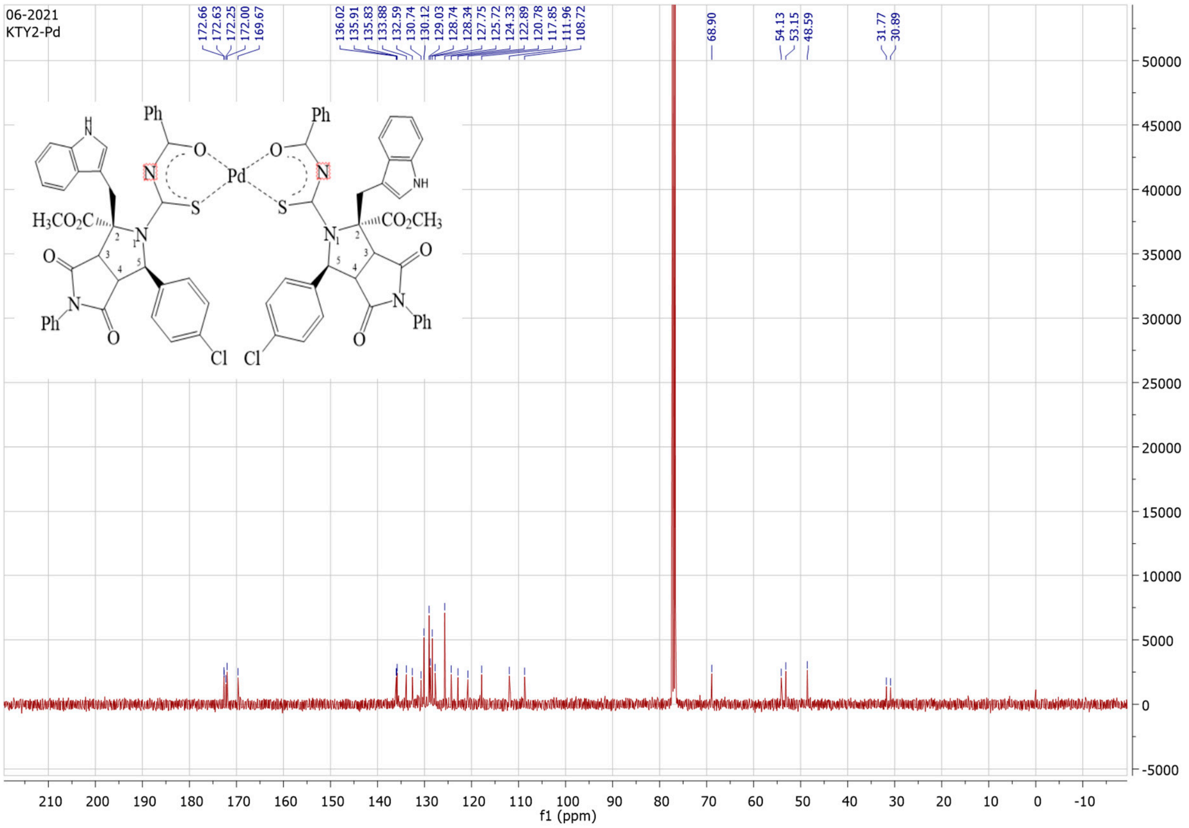

06-2021
KTY2-Pd
172.66
172.63
172.25
172.00
169.67
136.02
135.91
135.83
133.88
132.59
130.74
130.12
129.03
128.74
128.34
127.75
125.72
124.33
122.89
120.78
117.85
111.96
108.72
68.90
54.13
53.15
48.59
31.77
30.89
Ph
O
Pd
N
S
H_3CO_2C
CO_2CH_3
NH
Cl
f1 (ppm)
